# Long-term durability of alumina ceramic heads in THA

**DOI:** 10.1186/s12891-015-0703-2

**Published:** 2015-09-12

**Authors:** Nicholas A. Beckmann, Tobias Gotterbarm, Moritz M. Innmann, Christian Merle, Thomas Bruckner, J. Philippe Kretzer, Marcus R. Streit

**Affiliations:** Clinic for Orthopedics and Trauma Surgery, Center for Orthopedics, Trauma Surgery and Spinal Cord Injury, Heidelberg University Hospital, Schlierbacher Landstrasse 200A, 69118 Heidelberg, Germany; Institute of Medical Biometry and Informatics, University of Heidelberg, Im Neuenheimer Feld 305, 69120 Heidelberg, Germany

**Keywords:** Bearings, Ceramic, Polyethylene, Hip Arthroplasty, THA, Hip Replacement, THR

## Abstract

**Background:**

The optimal type of bearing for hip arthroplasty remains a matter of debate. Ceramic-on-polyethylene (CoP) bearings are frequently used in younger and more active patients to reduce wear and increase biocompatibility compared to Metal-on-Polyethylene (MoP) bearings. However, in comparison to metal heads, the fracture risk of ceramic heads is higher. In addition, ceramic head fractures pose a serious complication which often necessitates major revision surgery. To date, there are no long-term data (>20 years of follow-up) reporting fracture rates of the ceramic femoral heads in CoP bearings. The purpose of this research was to investigate long-term CoP fracture rate.

**Methods:**

We evaluated the clinical and radiographic results of 348 cementless THAs treated with 2^nd^ generation Biolox® Al_2_O_3_ Ceramic-on-Polyethylene (CoP) bearings consecutively implanted between January 1985 and December 1989. The mean age at implantation was 57 years. The patients were followed for a minimum of 20 years. At the final 111 had died, and 5 were lost to follow-up. The cumulative incidence of ceramic head fractures in the long-term was estimated using a competing risk analysis.

**Results:**

The cumulative incidence of ceramic head fracture after 22-years was estimated with a competing risk analysis at 0.29 % after 22-years (SE = 2.09 %; 95 % - CI: 0.03-1.5 %). The radiographic analysis revealed no impending failures at final follow-up.

**Discussion/Conclusion:**

The fracture rate of second-generation ceramic heads using a CoP articulation remains very low into the third decade after cementless THA.

## Background

Ceramic implants were introduced in Total Hip Arthroplasties (THA) in the 1970’s by Boutin [1] and were used for femoral heads, monolithic sockets and liners for acetabular cups [2]. The advantages were soon recognized. In comparison to metal components, ceramics show superior mechanical and tribological properties [2–4], they are very hard, scratch resistant, show increased wettability and have excellent biocompatibility [5]. In addition to the decreased wear of ceramic heads, literature indicates that they reduce fretting corrosion in comparison to metal heads [6]. However, with the early designs, it was found that ceramic-on-ceramic (CoC) bearings were associated with an increased fracture rate because of the brittleness of the ceramics.

Changes to the properties of the ceramics and changes to the design of the ceramic femoral head reduced the fracture rate. Use of a polyethylene (PE) liner rather than a ceramic acetabular liner reduced the fracture rate further [7]. In addition, Meftah et al. [8] have shown that ceramic-on-polyethylene (CoP) bearings result in less polyethylene wear than metal-on-polyethylene (MoP) bearings, a result confirmed by Dahl et al. [9] in their 10-year radiostereometric comparative follow-up of CoP with MoP bearings. It was anticipated that the properties of ceramic femoral heads in combination with PE liners would be particularly well suited to use in young people who have a long life expectancy and an active life style. Consequently they are often used in THA in this patient population.

Although ceramic head fracture occur infrequently today, in the event of a fracture occurring, the resulting revision surgery can prove very challenging, since the ceramic particles lodge into the surrounding soft tissue and can cause rapid revision implant failure if not adequately removed [10–12]. Short and mid-term follow-up studies reporting ceramic head fracture rates using CoP bearings have been published, and indicate good results [13, 14]. Long term follow up studies on fracture rates are few, with the longest follow up period being 18 years after initial arthroplasty and with only a small group of patients [14]. However, for evaluation of potential usefulness of a hip prosthesis using ceramic on polyethylene (CoP) bearings for placement in younger patients a long-term follow up is particularly important.

The goal of this study, therefore, is to evaluate long-term durability of ceramic heads in alumina on polyethylene articulations in cementless THA after a minimum follow-up of at least 20 years by using ceramic head fracture as the endpoint.

## Methods

We retrospectively evaluated the ceramic head failure rate in a consecutive series of 320 patients with 348 cementless THA implanted between January 1985 and December 1989 using 32 mm 2^nd^ generation Al_2_O_3_ ceramic femoral heads (Biolox®, Ceramtec GmbH, Plochingen, Germany; mean >99.7 Vol.-% Al_2_O_3_; Pore size 3.2 μm; E-modulus 410 ± 1 GPa – other than the pore size, these are similar properties to the currently used Biolox® Forte) articulating with ultra-high-molecular-weight polyethylene (UHMW-PE). The mean patient age at surgery was 57 years (range 13–81 years). The patient’s reasons for arthroplasty are listed in Table 1. All patients provided informed consent prior to study inclusion. The local institutional review and ethics boards (Ethikkommission der Medizinischen Fakultät Heidelberg) approved this study, which was carried out in accordance with the Declaration of Helsinki. The patient collective was previously reported on and evaluated with regard to the long-term survivorship of the femoral components [15].

**Table 1:** Diagnosis of patients undergoing THA

Diagnosis:	Number of hips:
Osteoarthritis	186 (53 %)
Congenital dislocation of hip	84 (24 %)
Avascular necrosis	39 (11 %)
Posttraumatic osteoarthritis	21 (6 %)
Rheumatoid arthritis	6 (2 %)
Neck fracture	5 (2 %)
Others	7 (2 %)
TOTAL	348 (100 %)

All of the titanium femoral stems (CLS® Spotorno® stem, Zimmer Inc., Warsaw, IN, USA) had a neck-shaft angle of 145°, a 14/16 taper and were implanted using a press-fit technique as previously described by Spotorno et al. [16].

All 348 hips received acetabular reconstruction with smooth uncemented threaded cups. Threaded, spherical, uncemented Mecron cups (Mecron GmbH, Berlin, Germany) were implanted into 222 of these hips (64 %) and 126 hips (36 %) received threaded, conical, uncemented Weill rings (Zimmer Inc., Warsaw, IN, USA). The acetabula were prepared as recommended by the respective manufacturer.

23 surgeons performed the implantations at our institution during the designated time period, using either a modified Watson-Jones or a lateral transgluteal approach as described by Bauer et al. [17] with the patient in the supine position. The Al_2_O_3_ femoral heads were carefully placed onto the 14/16 femoral CLS Spotorno® tapers after thorough cleansing of the taper in order to reduce the risk of damage to the ceramic material. The patients were advised to limit their physical activity to partial weight bearing for six weeks after surgery, after which full mobilization and weight bearing was permitted. The patients were then followed up after surgery at regular intervals of 3 months, 6 months, 1 year, and every 5 years thereafter.

### Radiographic evaluation

Standard anteroposterior radiographs (ap) of the pelvis and hip and frog leg lateral radiographs of the hip were taken. Two independent observers (NAB, MRS) evaluated the ceramic heads for irregularities and signs of fracture.

### Statistical analysis

The cumulative fracture incidence of the Al_2_O_3_ femoral heads over the long-term was estimated using a competing risks analysis, which was chosen to compensate for possible competing risks through failure of the prosthesis for any other cause [18, 19]. Cases were censored either at time of patient death, at time of femoral head revision due to acetabular or stem failure or at loss to follow-up. The endpoint used was failure of the ceramic head or revision due to ceramic head failure. SPSS® Version 22.0 (SPSS Inc, Chicago, IL, U.S.A.), SAS® Version 9.4 (SAS Institute Inc., Cary, NC, USA) and Graphpad Prism® Version 6.01 (Graphpad Software, La Jolla, CA, U.S.A.) were used to record and analyze the data.

## Results

**Fig. 1:**
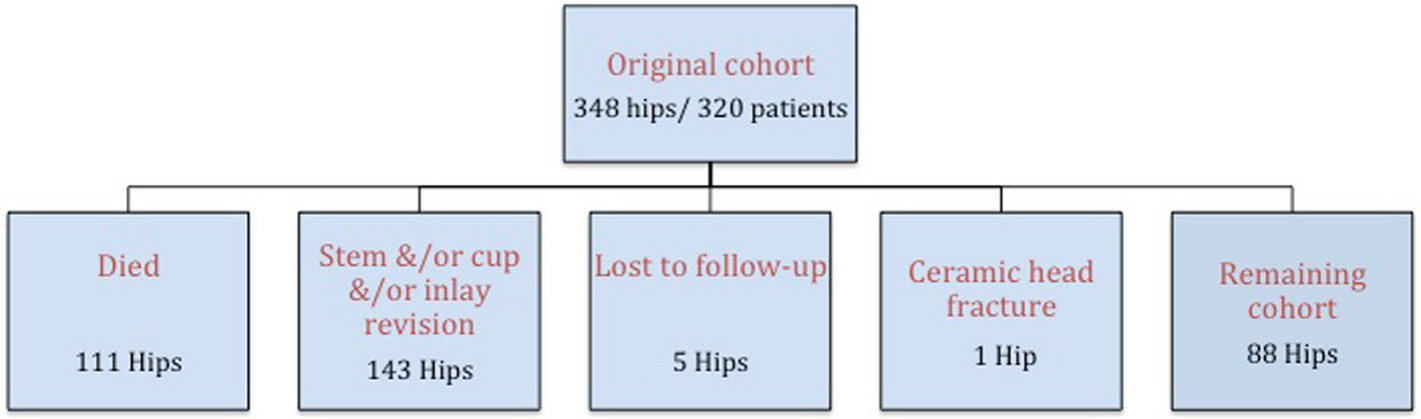
Flow chart displaying distribution of hips at mean 22-year final follow-up

**Fig. 2:**
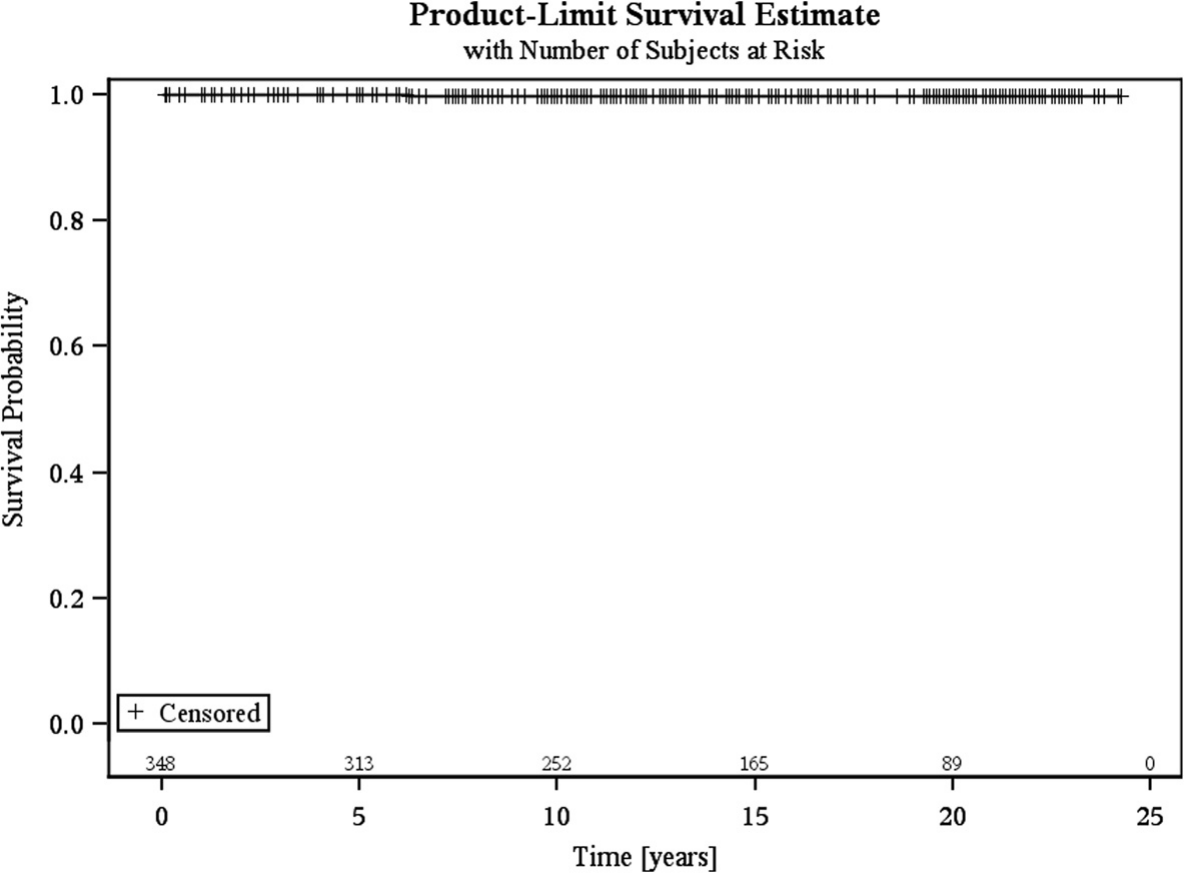
Competing risks survivorship curve showing the product-limit survival estimate after 22-years (ceramic head failure incidence = 0.29 % after 22-years (SE = 2.09 %; 95 % - CI: 0.03-1.5 %)

An overview of the results can be seen in Figs. 1 and 2.

At the final follow-up (mean 20-year, range 20–25 year follow-up) of the 348 hips in the 320 patient collective, 111 hips (32 %) had died. An additional 5 hips (1 %) were lost to follow-up. At the time of latest follow-up, 143 hips had been revised due to reasons other than ceramic head failure. In total, 132 acetabular components and 41 femoral components were revised, and in 55 cases, an isolated revision of the polyethylene bearing and the modular femoral head was performed for wear. In all of these cases involving acetabular or femoral component revision, the modular femoral head was exchanged. One hip was revised after fracture of the alumina femoral head 6.25 years after surgery. The remaining 88 hips were radiographically evaluated at a mean follow-up time of 22 years (range, 20–25 years) and appeared to be intact (see Flow Chart Fig. 1).

The competing risks analysis revealed a cumulative incidence of ceramic head fractures at 0.29 % after 22-years (SE = 2.09 %; 95 %-CI, 0.03-1.5 %) (Fig. 2).

The patient suffering the ceramic head fracture was a woman who had received a CLS stem, Biolox head, and Mecring Cup at 55 years of age due to osteoarthritis. The implantation was carried out through a Watson-Jones approach. The first several years after implantation were without note, and the patient was pain-free. 5 ½ years after implantation she began complaining of recurring pain in the groin and trochanter; no apparent radiographic or szintigraphic signs of loosening were visible. 6.25 years after the implantation she noted a sudden increase in pain, at which point the ceramic head fracture was confirmed radiographically. The intraoperative revision procedure showed a comminuted ceramic head, with substantial signs of soft tissue metallosis, as well as loosening of both the stem and cup. The patient was subsequently treated with a total synovectomy, and total revision surgery with a cemented Weller stem, Mueller revision ring with a cemented standard PE cup (Aesculap®) and autologous spongiosa transplantation to fill defective bone stock.

**Fig. 3:**
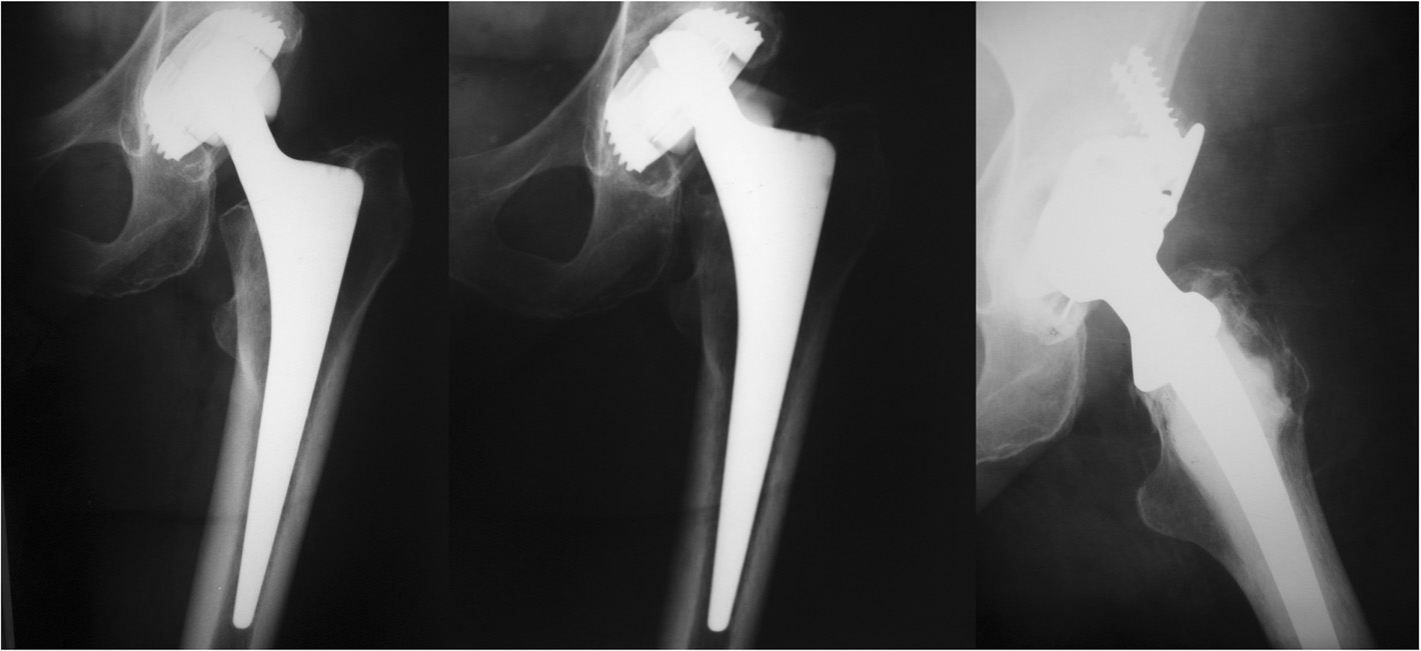
Radiographs of the hip with the fractured ceramic head. Left: directly post-OP: Middle: Fracture of ceramic head at 6.25 years postop; Right: After revision arthroplasty

Standard radiographic evaluation at the final follow-up showed no signs of failure or fracture of the femoral head in the unrevised hips evaluated radiographically. The hip revised for ceramic femoral head fracture at 6.25 years is shown in Fig. 3.

## Discussion

Hip arthroplasty is being carried out with increasing frequency [20] in younger and more active patients [21] and is considered one of the most successful procedures in orthopaedic surgery [22]. Despite excellent long-term THA results, the optimal bearing for hip arthroplasty remains unclear. The most common cause for failure of a total hip arthroplasty is aseptic loosening due to wear of the articulating surfaces causing osteolysis [23–25]. Although ceramic on ceramic bearings have superior tribological and biological characteristics according to Fisher et al. [26], there is the potential risk of fracture of the ceramic components. This concern was assuaged with the use of polyethylene liners (CoP). These not only significantly reduced the wear rate compared to metal on polyethylene (MoP) bearings [9, 27] resulting in decreased osteolysis and decreased loosening [5, 27], they also significantly reduced the fracture rate compared to CoC [7]. Further improvement and significant decrease of the wear rate was achieved by the introduction of a cross-linked polyethylene liner [28–30]. However, there is still insufficient long term data from any large series on ceramic head fracture rates into the third decade after cementless THA although the CoP bearing is frequently used in THA, particularly in younger patients.

The present study is the first to evaluate the long-term fracture-free survival of ceramic-on-polyethylene bearings into the third decade. Our study represents a large cohort of patients with the longest follow up period who underwent THA with CoP bearings in one institution. It confirms short- and midterm follow-up studies that have reported a low fracture risk. In our series we found only one case of Al_2_0_3_ head fracture out of 348 THA’s resulting in a cumulative fracture incidence of 0.29 % in the competing risk analysis at 22 years.

Table 2 summarizes the literature on ceramic femoral head fracture rates after THA with CoP. Earlier studies on ceramic head fractures with large patient cohorts have been reported, but only with short or midterm follow-ups. Fritsch et al. [3] reported on 1341 hips treated with Ceramic-on-Polyethylene bearings with an average of 6 years follow-up period. They noted one ceramic head fracture among their collective, or 0.07 %. Hannouche et al. [31] noted 2 fractured ceramic heads in their population of 1200 hips (or 0.17 %) with alumina ceramic heads and polyethylene liners (of a total of 5500 hips treated with alumina components). However, these authors did not identify the follow-up period. Fayard et al. [32] reported on their patient collective involving 120 hips with CoP bearings with a mean FU of 8.4 years and 2 ceramic head fractures, or 1.7 %, were noted. In contrast to these very small fracture rates are the numbers reported by Callaway et al. [33], who found 4 ceramic head fractures, or 2.2 %, in their 184 CoP treated hips. No follow up times were specified, however; consequently, the longevity of the bearing couple cannot be assessed. There are only two prior studies that have reported long term results with CoP bearings and these studies have significantly smaller populations. Yoon et al. [34] reported 15–20 year results on 43 uncemented hips with CoP bearings. None of these patients suffered ceramic fractures. Urban et al. [14] reported results on 64 cemented hip arthroplasties with a 32 mm Alumina femoral head on an all-polyethylene cup after a 17–21 year follow up. No ceramic head fractures occurred in their patients.

**Table 2:** Overview of the literature on fracture in Ceramic-on-Polyethylene Bearings

Hip Prosthesis			Author	Year of publication	Mean FU	Mean patient age (years)	# of Hips	lost to FU	Fracture ceramic head	% Ceramic Fracture
Femoral Head	Liner	Fixation			Years	(*Range*)				
Alumina, Biolox delta, (DePuy)	Highly x-linked PE (Crossfire®, Stryker®)	Uncemented	Beaupre [37]	2013	5 years	53.6	44	10	0	0
28-mm Alumina, Biolox Forte, (DePuy)	PE Depuy, Enduron	Uncemented	Wang [27]	2013	10 year	51.5 (36–59)	22	?	0	0
28 mm Alumina, (Ceramtec)	conventional UHMW PE	Uncemented	Meftah [8]	2013	17 (+/− 1.7)	55 (+/−9)	31	0	0	0
26x32mm; 37x36mm Alumina, Biolox delta	Highly x-linked PE (Trident X3, Stryker)	Uncemented	Meftah [38]	2011	2.9 (+/−0.5)	60.9 (+/− 8.9)	72	0	0	0
28 mm Alumina	50 UHMW PE Sandwich, 16 Ceramic Inlay	Uncemented cups; cemented stems	Kircher [39]	2009	1	56.7 (+/−8.5)	47	?	1	2.1
Alumina, Biolox	84 Alumina (Biolox), 43 PE	Uncemented	Yoon [34]	2008	17.1	41 (20–64)	43	?	0	0
28 mm Alumina, (Biomet)	PE	Uncemented	Fayard [32]	2006	8.4 (+/−2.5)	66.6 (+/−10.7)	102	?	2	2.0
32 mm Alumina, (Biomet)	UHMW-PE	Uncemented	Legenstein [13]	2006	17 years)	61.2	119	51	0	0
Alumina	PE	Not reported	Hannouche [31]	2003	?	?	1200	?	2	0.17
32 mm Alumina, (Feldmuhle)	UHMW PE, (DePuy)	Cemented	Urban [14]	2001	18.2 (17.2-21.3)	69 (51–84)	64	41	0	0
Alumina, Biolox	PE (various manufacturors)	Both cemented & uncemented	Fritsch [3]	1996	6	?	1341	?	1	0.07
28 mm Alumina, Biolox	PE	Both cemented & uncemented	Callaway [33]	1995	?	?	184	?	4	2.2
*2* ^*nd*^ *Gen. 32 mm, Biolox*	*UHMW PE*	*Uncemented*	*Current study*	*2015*	*22*	*57 (13–81)*	*348*	*5*	*1*	*0.29*

If one were to assume that the risk of a femoral head fracture is 0 for MoP hips at any point in time (a realistic assumption), then the number of hips needed to treat to avoid one femoral head fracture is 1/(0.0029) = 344.8. In other words, 344.8 patients need to get a MoP hip instead of a CoP hip in order to prevent 1 head fracture. This must, however, be weighted against the increased wear rate seen in MoP over CoP as had been shown in clinical studies by Meftah et al. [8] and Wang et al. [27].

There are several limitations in our study. This is a retrospective uncontrolled study with a possible selection bias: our collective of 348 patients who had received total hip arthroplasty with the aforementioned uncemented prostheses with CoP bearings represented 34 % of the total number of patients that received THA at our institution during the time period examined (January 1985 – December 1989). These implants were selected by the operating surgeon at the time of the procedure. The patient collective tended to be younger and more active than the remaining 66 % of patients operated on during this time period and therefore had a potentially increased risk of complications including ceramic head fractures. Furthermore, the implanted materials/components are not state-of-the-art and some are no longer being used due to high known failure rates, which may have increased the wear related revisions seen as well as the overall revision rate. For example, 222 patients (64 % of our collective) had received a Mecring Mecron® acetabular cup, which has been shown in other studies to have a high failure rate [35], and in our cohort led to an increased revision rate of the acetabular component (38 %) [15] and femoral head prior to 20 years, and therefore a decreased number of heads at risk after more than 20 years [18, 36]. Since the introduction of the second generation Al_2_O_3_ Biolox® ceramic femoral heads and conventional ultra-high-molecular-weight polyethylene (UHMW-PE) components that were used in our patients, many additional advances have been made which should further decrease the failure rate of alumina ceramic femoral heads.

## Conclusion

Although we used an older 2nd generation Biolox® design that is not currently considered “State of the Art”, the long-term fracture rate documented with CoP bearings was very low. In combination with the previously published decreased wear rate [8, 27], CoP bearings could represent a promising alternative to metal-on polyethylene bearings especially in young people. Future long-term studies comparing CoP bearings composed of newer materials to metal-on polyethylene bearings may provide additional support for their use.

## Abbreviations

THA: Total hip arthroplasty
THR: Total hip replacement
CoP: Ceramic-on-polyethylene
MoP: Metal-on-polyethylene
CoC: Ceramic-on-ceramic
CI: Confidence interval
SE: Standard error
PE: Polyethylene
UHMW-PE: Ultra-high-molecular-weight-polyethylene
